# Single nucleotide polymorphisms associated with methotrexate-induced nausea in juvenile idiopathic arthritis

**DOI:** 10.1186/s12969-021-00539-9

**Published:** 2021-04-01

**Authors:** Nini Kyvsgaard, Torben Stamm Mikkelsen, Thomas D. Als, Anne Estmann Christensen, Thomas J. Corydon, Troels Herlin

**Affiliations:** 1 Pediatric and Adolescent Medicine, Department of Clinical Medicine, Aarhus University Hospital, Palle Juul-Jensens Boulevard 99, 8200 Aarhus N, Denmark; 2 Department of Biomedicine, Aarhus University, Aarhus, Denmark; 3 Department of Pediatric Rheumatology, H.C. Andersen’s Children’s Hospital, Odense University Hospital, Odense, Denmark; 4 Department of Ophthalmology, Aarhus University Hospital, Aarhus, Denmark

**Keywords:** JIA, Juvenile idiopathic arthritis, SNPs, Single nucleotide polymorphisms, MTX, Methotrexate

## Abstract

**Background:**

- ***Context:*** Methotrexate (MTX) is a cornerstone in the treatment of juvenile idiopathic arthritis (JIA). MTX treatment is commonly associated with nausea. Large inter-individual variation exists in the level of MTX-induced nausea, possibly due to genetic factors.
- ***Purpose:*** To investigate whether MTX-induced nausea was associated with single nucleotide polymorphisms (SNPs) in genes encoding MTX-transporter proteins, a MTX metabolizing enzyme and a nausea receptor.

**Findings:**

- ***Methods:*** Children aged ≥9 years treated with MTX for JIA were eligible. MTX-induced nausea was registered by the children’s completion of a nausea diary (min. 7 days) and the parents’ completion of the MTX intolerance severity score (MISS). The selected SNPs were: *SLCO1B1* (rs4149056; rs4149081), *SLCO1B3* (rs2117032), *SLC19A1* (rs1051266), *ABCC2* (rs2273697; rs3740066; rs717620), *ABCB1* (rs2032582; rs1045642), *MTHFR* (rs1801131, rs1801133), *HTR3A* (rs1062613; rs1985242; rs1176713) and *HTR3B* (rs1176744).
- ***Result:*** Enrolled were 121 JIA patients (82 girls: 39 boys) with a median age of 13.3 years (IQR: 11.3–15.1). The median MTX dose was 9.7 mg/m^2^/week (IQR: 9.0–10.9). The median MTX treatment duration prior to enrolment was 340 days (IQR: 142–766). The SNP analysis was available for 119 patients. MTX intolerance was associated with the genotype distribution of rs1801133 (*MTHFR*) (*p* = 0.02). There was no additive effect of the minor alleles for any of the selected SNPs, nor any significant haplotype associations.

**Conclusion:**

- ***Summary:*** MTX-induced nausea may be influenced by genetic polymorphisms in a MTX metabolizing enzyme (rs1801133; MTHFR).
- ***Implications:*** Further analyses involving inclusion of larger cohorts are needed to understand the impact of SNPs on MTX-induced nausea in JIA.

## Findings

### Background and hypothesis

Methotrexate (MTX) continues to have a key role in the treatment of children with juvenile idiopathic arthritis (JIA) [1–3]. MTX treatment is commonly associated with nausea. Large inter-individual variation exists in the level of MTX-induced nausea among children with JIA. From mild discomfort to a level of MTX-induced nausea that affects health-related quality of life to such an extent that premature termination is necessary [4–6]. Genetic factors are thought to influence the inter-individual variation in MTX-induced nausea.

**Fig. 1:**
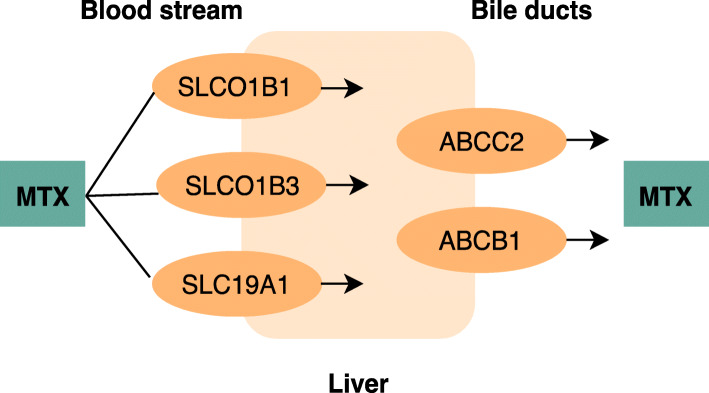
Illustration of methotrexate transporter proteins in the liver. **MTX**; Methotrexate. **SLCO1B1, SLCO1B3, SLC19A1, ABCC2 and ABCB1**; Methotrexate transporter proteins

MTX is mainly excreted through the kidneys, but the drug also undergoes enterohepatic circulation [7, 8]. The level of enterohepatic circulation is thought to be associated with the level of MTX-induced gastrointestinal side effects, including nausea [9]. Single nucleotide polymorphisms (SNPs) in genes encoding MTX transporter proteins may alter the function of these transporter proteins and hence affect the enterohepatic circulation of MTX and the level of MTX-induced gastrointestinal side effects [10–13] (Fig. 1).

**Fig. 2:**
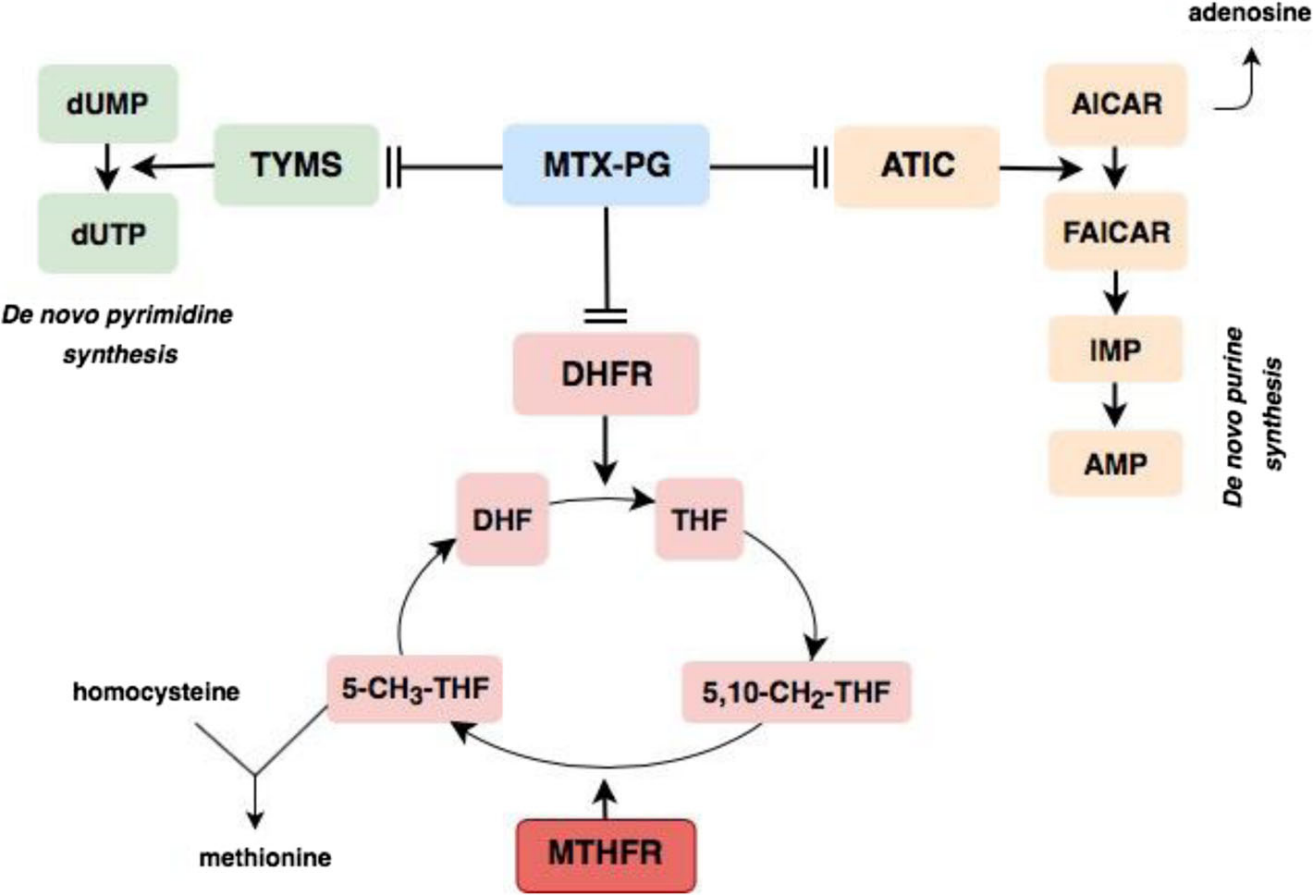
Illustration of the intracellular effects of methotrexate-polyglutamates including the role of the MTHFR enzyme. **dUMP**; deoxyuridine monophosphate. **dTMP**; deoxythymidine monophosphate. **TYMS**; thymidylate synthase. **MTX-PG**; methotrexate polyglutamates. **DHFR**; dihydrofolate reductase. **DHF**; dihydrofolate. **THF**; tetrahydrofolate. **5-CH**_**3**_**-THF**; 5-methyltetrahydrofolate. **5,10-CH**_**2**_**-THF**; 5,10-methylenetetrahydrofolate. **MTHFR**; methylenetetrahydrofolate reductase. **ATIC**; AICAR formyltransferase. **AICAR**; 5-aminoimidazole 4-carboxamide ribonucleotide. **FAICAR**; 10-formyl AICAR. **IMP**; inosine monophosphate. **AMP**; adenosine monophosphate

MTX inhibits folate pathway enzymes including methylentetrahydrofolatereductase (MTHFR) [14]. This leads to an altered folate homeostasis and specifically the reduced MTHFR activity causes accumulation of homocysteine (Fig. 2), which has been hypothesized to be associated with MTX-induced gastrointestinal adverse events [15]. Specific SNPs in the gene encoding MTHFR have been shown to cause a decreased enzyme-activity [16, 17] and studies in smaller JIA cohorts have indicated that these SNPs may be associated with MTX-induced adverse effects [18, 19].

**Fig. 3:**
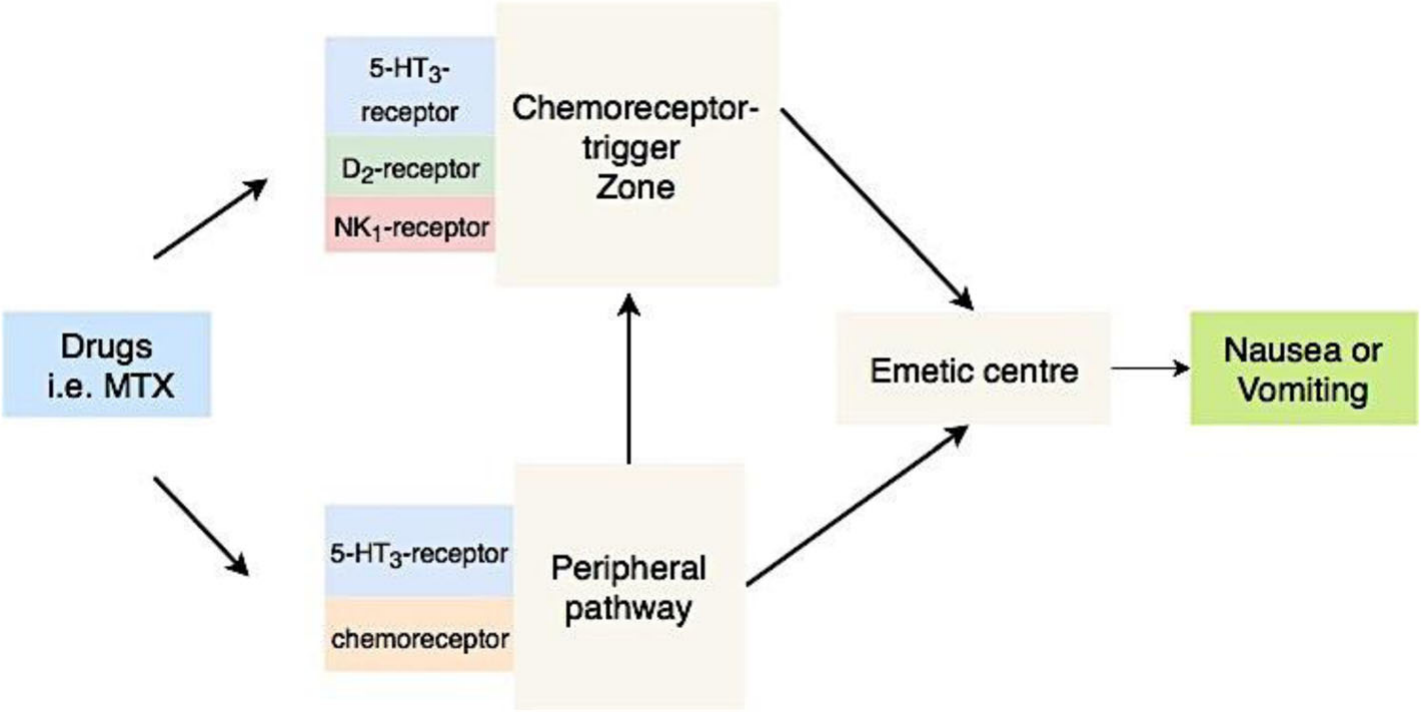
Illustration of pathways mediating methotrexate-induced nausea. **5-HT3-receptor;** 5-hydroxytryptamine type 3 receptor. **D**_**2**_**-receptor**; Dopamine type 2 receptor. **NK**_**1**_**-receptor**; Neurokinin type 1 receptor. **MTX;** Methotrexate

Nausea-inducing medicine can activate the 5-hydroxytryptamine type 3 (5-HT_3_)-receptor placed in the gastrointestinal tract and the medulla oblongata, which causes a signal to be transmitted to the emetic center in the central nervous system [20, 21] (Fig. 3). Studies have shown that SNPs in *HTR3A* and *HTR3B* encoding subunits of the 5-HT_3_-receptor are associated with impaired receptor function and nausea in adult cancer patients [22, 23]. Hence children with SNPs affecting the function of these genes may be more prone to experience MTX-induced nausea.

The objective for this study was to investigate whether MTX-induced nausea was associated with selected SNPs in candidate genes encoding MTX transporter proteins (*SLCO1B1, SLCO1B3, SLC19A1, ABCB1, ABCC2*), the MTHFR enzyme (*MTHFR*) and the 5-HT_3_-receptor (*HTR3A, HTR3B*), in children with JIA.

## Methods

### Study population

**Fig. 4:**
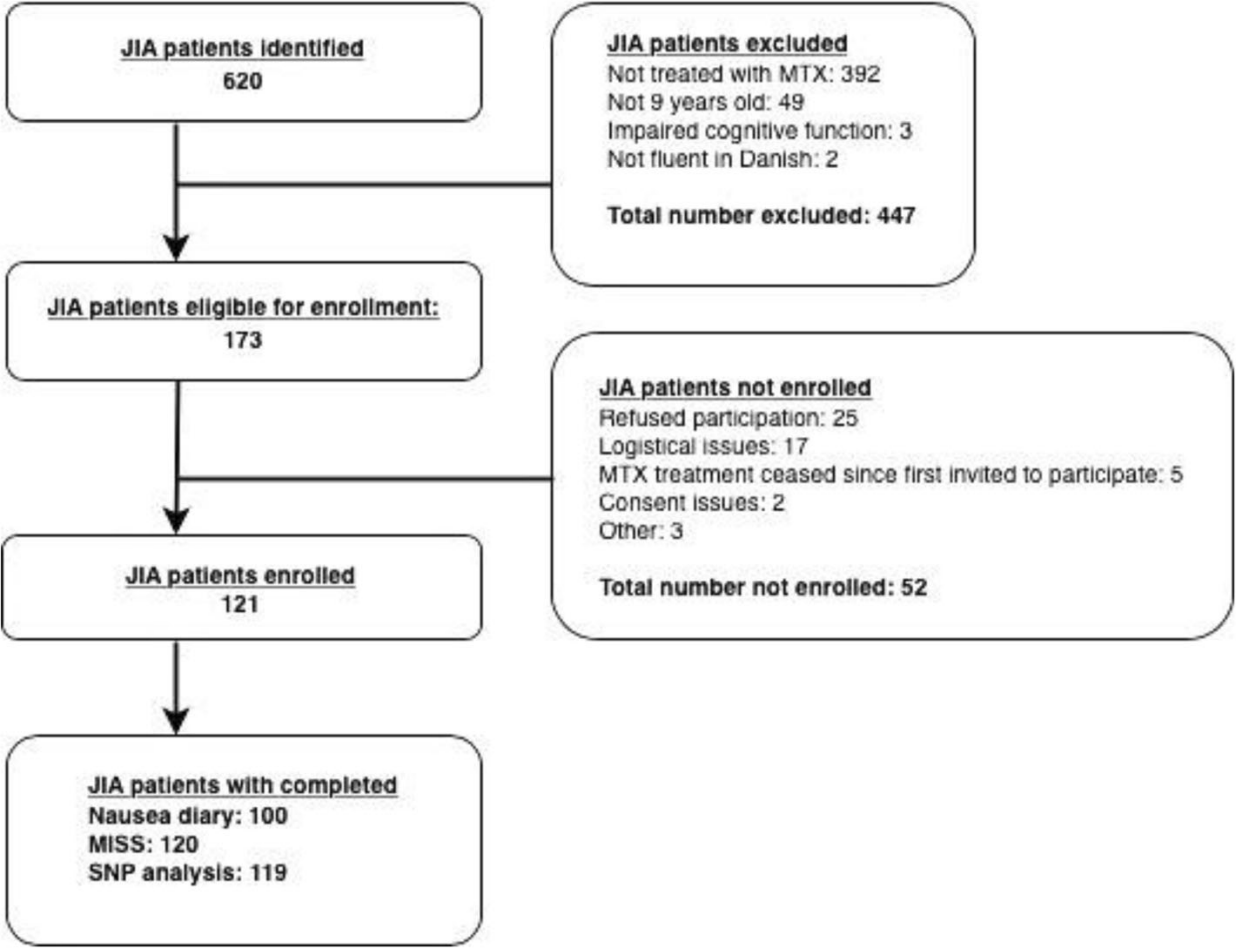
Flowchart of the enrollment and exclusion of patients. **MTX**; Methotrexate. **MISS**; Methotrexate intolerance severity score. **SNP**; Single nucleotide polymorphism

The study population was composed of children diagnosed with JIA according to the International League of Associations for Rheumatology criteria, treated with methotrexate and aged nine years or above. Children were excluded if cognitively impaired or not fluent in Danish (Fig. 4). All children were followed at our pediatric rheumatology outpatient clinics and enrolled from December 2013 until July 2016.

### Phenotype

MTX-induced nausea was determined using two outcome measures previously described [24]: a Danish adaption of the methotrexate intolerance severity score (MISS) [6] and a nausea diary. The outcome measures were developed in electronic versions. The MISS was completed on the day of enrolment by the patients’ parents and they were instructed to focus on their child’s current state of MTX intolerance. The nausea diary was intended to be completed daily by the children for 28 consecutive days following enrolment. It consisted of categorically structured questions covering whether the child had experienced nausea and if so, its timely relation to MTX.

Phenotypes were established based on the two outcome measures. As defined by the MISS development paper [6], a child was categorised as MTX_intolerant_ if the MISS total score was six or above with at least one point for an anticipatory and/or associative and/or behavioral symptom; otherwise categorised as MTX_tolerant_. The phenotype “MTX-nausea” was defined based on the nausea diary. It included children with a nausea diary illustrating a nausea pattern timely related to MTX administration, i.e. the child had to report a clear coherent pattern of nausea on the day of MTX administration and/or the day after; and at least have completed seven diary entries.

### DNA extraction

On the day of enrolment a blood sample was drawn for the SNP analysis. DNA was extracted from EDTA blood by salt precipitation with triton lysis buffer (Tris 10 mM, MgCl_2_ 5 mM, Triton X-100 1% and Sucrose 0.32 M), nucleus lysis buffer (NaCl 75 mM and EDTA 24 mM), saturated NaCl (6 M), isopropanol and Te-buffer (Tris-HCl 10 mM, EDTA 1 mM, pH 7.5). DNA concentration and purity was measured by a Nanodrop spectrophotometer.

### SNP analysis

For the SNP analysis an amplicon with the specific SNP was created, PCR amplified and then sequenced by Sanger Sequencing. The analysis was performed at Eurofins Genomics using BigDye terminator cycle sequencing chemistry (version 3.1/3.0; Thermo Fisher Scientific), peqStar 96 HPL (PEQLAB Biotechnologie GMBH) and/or GeneTouch (Biozym Scientific GmbH) thermal cyclers, ABI3730xl capillary sequencers (Thermo Fisher Scientific) and GoTaq HotStart Green MasterMix (Promega). The quality scoring of the sequences was based on the quality value at the base-position, the peak-appearance, the overall signal intensity and quality of the sequence, the relation of forward and reverse sequence, and the identification of unspecific/artificial background signals or secondary PCR products and their subsequent signal. The quality scoring was done manually by Eurofins Genomics.

### SNP selection

Within the specified candidate genes SNPs were selected based on clinical and genetic criteria. The SNPs should preferably have been proven clinically significant for gastrointestinal intolerance or toxicity, or proven phenotypically significant in animal studies. Further, they should have been studied in a relevant study population, have a reasonable minor allele frequency, a functional significance and preferably cause an amino acid change. The GTExportal.org was used to see whether the relevant gene section is expressed in relevant tissue. SNPsnap Broad Institute was used to see whether SNPs were in linkage disequilibrium.

### Statistics

All statistical analyses were performed in STATA-13, PLINK 1.9 or haplo.stats R-package. Data on demographics and MTX treatment were analysed with Wilcoxon rank-sum test, chi-squared test/Fisher’s exact test or Student’s T-test, where applicable. The associations between SNP genotypes and phenotypes were analysed with chi-squared test/Fisher’s Exact test in STATA-13. Logistic regression (PLINK 1.9) was used to analyze the additive effects of the minor alleles. The analyses were filtered for Hardy-Weinberg equilibrium and took into account whether SNPs had more than two possible alleles present in the study population. Haplotype based association tests were performed for the two phenotypes – the method implemented in the *R*-package haplo.stats (https://CRAN.R-project.org/package=haplo.stats) and described by Schaid et al. [25] was used. The method allowed missing alleles and was developed for unrelated subjects, marker-alleles with unknown phase and ambiguous haplotypes. A progressive insertion algorithm was used to compute maximum likelihood estimates of haplotype probabilities. Additive models were chosen for all datasets.

## Results

The study population encompassed 121 patients. Figure 4 illustrates a flowchart of the exclusion and enrolment of patients. There was missing data for the two outcome measures due to patients or parents never completing an entry, despite having consented to participation. Due to laboratory problems at enrolment SNP data was missing for two patients.

### Demographics, MTX details

Table 1 shows demographic data, details of the MTX treatment and the distribution of children within the phenotype subgroups. The nausea diary was completed at least one day by 100/121 (83%) of the children. The 77 children who had completed at least seven diary entries were included in the genetic analysis for this phenotype. A total of 8/77 (10%) of the children reported no nausea in all their diary entries.

**Table 1:** Demographics, methotrexate treatment and the phenotype subgroups

	JIA
**JIA patients,** ***n***	121
**Girls: Boys,** ***n***	82:39
**Caucasian: not Caucasian,** ***n***	117:4
**Age at enrolment (years)**	13.3 (11.3–15.1)
**Duration of MTX treatment (days)**	340 (142–766)
**MTX**_**o**_**: MTX**_**SC**_**,** ***n***	45:76
**MTX dose (mg/m**^**2**^**/week)**	9.7 (9.0–10.9)
**Patients with completed MISS,** ***n***	120
**MISS total score (0–36)**	8 (3–14)
**MTX**_**intolerant**_ **subgroup**^**1**^**,** ***n***	73
**MTX**_**tolerant**_ **subgroup,** ***n***	47
**Patients with** ≥ **7 completed diary entries,** ***n***	77
**“MTX-nausea” subgroup**^**2**^**,** ***n***	56

Data on demographics, details of the methotrexate (MTX) treatment and the distribution of patients in the phenotype subgroups

*Values are expressed as median (IQR) unless otherwise stated. MTX*_*SC;*_ subgroup treated with MTX subcutaneously*,* MTX_o_ *subgroup treated with MTX orally*

^1^The MTX_intolerant_ subgroup: children with a total score of the Methotrexate Intolerance Severity Score (MISS) ≥6 and at least 1 point in the anticipatory and/or associative and/or behavioral symptoms. Otherwise categorised as MTX_tolerant_

^2^The “MTX-nausea” subgroup: children with a nausea diary illustrating a nausea pattern timely related to the MTX administration

All patients were treated with folic acid supplement (5 mg) the day after MTX administration. Antiemetic medicine was prescribed for 41/121 (34%) of the children at enrolment.

### SNPs in MTX transporter proteins

The selected SNPs within the candidate genes were: SLCO1B1 (rs4149056; rs4149081), SLCO1B3 (rs2117032), SLC19A1 (rs1051266), ABCC2 (rs2273697; rs3740066; rs717620), ABCB1 (rs2032582; rs1045642). No significant associations were found between the genotype distribution for these SNPs and the phenotype subgroups (Table 2), nor any significant additive effect of the minor alleles on either of the two phenotypes (MTX_intolerant_ or “MTX-nausea”) (Table 3 and Table 4). No significant haplotype associations with either phenotype were found (Table 5 and Table 6).

**Table 2:** Genotypes for the selected single nucleotide polymorphisms

Gene	SNP	Ancestral Allel	Genotypes	Total, ***n***	MTX _**intolerant**_^**1**^	MTX tolerant	***p***-value	MTX Nausea^**2**^	Controls	***p***-value
***ABCC2***	**rs2273697**	**G**	**GG**	74	44	29	χ^2^ = 0.05	32	14	0.81
			**GA**	32	20	12	*p* = 0.98	16	5	
			**AA**	13	8	5		8	2	
***ABCC2***	**rs3740066**^**3**^	**G**	**GG**	38	23	15	0.74	19	7	1.00
			**GA**	65	38	27		29	12	
			**AA**	15	10	4		7	2	
***ABCC2***	**rs717620**	**G**	**GG**	73	47	26	0.64	35	13	1.00
			**GA**	39	21	17		18	7	
			**AA**	7	4	3		3	1	
***ABCB1***	**rs2032582**	**G**	**GG**	38	23	15	0.28	18	6	0.95
			**GT**	58	34	24		27	10	
			**GA**	2	0	2		1	1	
			**TA**	3	3	0		2	1	
			**TT**	18	12	5		8	3	
***ABCB1***	**rs1045642**	**C**	**CC**	18	9	9	**χ**^2^ = 1.34	8	4	0.82
			**CT**	68	44	24	*p* = 0.51	34	11	
			**TT**	33	19	13		14	6	
***SLCO1B1***	**rs4149056**	**T**	**TT**	84	49	34	0.86	39	15	1.00
			**TC**	32	21	11		16	6	
			**CC**	3	2	1		1	0	
***SLCO1B1***	**rs4149081**	**G**	**GG**	80	47	32	0.86	38	14	1.00
			**GA**	36	23	13		17	7	
			**AA**	3	2	1		1	0	
***SLCO1B3***	**rs2117032**	**C**	**CC**	17	12	5	χ^2^ = 2.16	10	4	0.63
			**CT**	53	28	24	*p* = 0.34	23	6	
			**TT**	49	32	17		23	11	
***SLC19A1***	**rs1051266**^**3**^	**G**	**GG**	33	18	15	χ^2^ = 0.76	17	6	0.89
			**GA**	66	42	24	*p* = 0.68	28	12	
			**AA**	19	11	7		10	3	
***HTR3A***	**rs1062613**	**C**	**CC**	80	52	28	0.13	36	16	0.58
			**CT**	37	20	16		19	5	
			**TT**	2	0	2		1	0	
***HTR3A***	**rs1985242**	**A**	**AA**	7	3	4	0.23	4	1	0.34
			**AT**	55	30	24		28	7	
			**TT**	57	39	18		24	13	
***HTR3A***	**rs1176713**	**C**	**CC**	6	3	3	0.68	3	1	0.18
			**CT**	37	21	16		19	3	
			**TT**	76	48	27		34	17	
***HTR3B***	**rs1776744**	**T**	**TT**	50	30	20	0.09	24	10	0.58
			**TG**	58	32	25		27	8	
			**GG**	11	10	1		5	3	
***MTHFR***	**rs1801131**^**3**^	**A**	**AA**	52	34	18	**χ**^2^ = 0.93	24	9	0.29
			**AC**	53	30	22	*p* = 0.63	21	11	
			**CC**	13	7	6		10	1	
***MTHFR***	**rs1801133**	**C**	**CC**	58	33	25	0.02	27	10	1.00
			**CT**	51	29	21		24	10	
			**TT**	10	10	0		5	1	

The genotype distribution for the selected single nucleotide polymorphisms (SNPs) – including the distribution of genotypes within the phenotype subgroups defined by the methotrexate intolerance severity score and the nausea diary

*SNP; Single Nucleotide Polymorphism*

^**1**^The MTX_intolerant_ subgroup: children with a total score of the Methotrexate Intolerance Severity Score (MISS) ≥6 and at least 1 point in the anticipatory and/or associative and/or behavioral symptoms (*n* = 72). Otherwise categorised as MTX_tolerant_ (*n* = 46)

^**2**^The “MTX-nausea” subgroup: children with a nausea diary illustrating a nausea pattern timely related to the MTX administration (*n* = 56). The remaining children grouped as “Controls” (*n* = 21)

^3^SNP variant not available for one child

**Table 3:** Single nucleotide polymorphisms in association with the phenotype “MTX intolerance”

	Gene	SNP	Alleles, Major > minor	MTX_**intolerant**_^**1**^ Allel count	MTX_**tolerant**_ Allel count	OR (95% CI)	***p***	Chr.	Genomic position	Consequence
**Hepatic**	**ABCC2**	**rs2273697**	G > A	36	22	1.05 (0.61–1.80)	0.87	10	99,804,058	Missense
**Efflux**	**ABCC2**	**rs3740066**^**2**^	G > A	58	35	1.15 (0.64–2.07)	0.64	10	99,844,450	Missense
**Transporter**	**ABCC2**	**rs717620**	G > A	29	23	0.77 (0.42–1.41)	0.40	10	99,782,821	5’UTR variant
	**ABCB1**	**rs2032582**	G > T	61	34	1.28 (0.73–2.24)	0.39	7	87,531,302	Missense
			G > A	3	2	0.96 (0.15–5.96)	0.96			
	**ABCB1**	**rs1045642**	T > C	62	42	0.88 (0.49–1.57)	0.67	7	87,509,329	Synonymous
**Hepatic**	**SLCO1B1**	**rs4149056**	T > C	25	13	1.28 (0.62–2.66)	0.51	12	21,178,615	Missense
**Influx**	**SLCO1B1**	**rs4149081**	G > A	27	15	1.19 (0.59–2.43)	0.62	12	21,225,087	Intron variant
**Transporter**	**SLCO1B3**	**rs2117032**	T > C	52	34	0.97 (0.57–1.64)	0.90	12	20,921,188	Down stream 3’UTR
	**SLC19A1**	**rs1051266**^**2**^	G > A	64	38	1.20 (0.67–2.13)	0.54	21	45,537,880	Missense
**Nausea**	**HTR3A**	**rs1062613**	C > T	20	20	0.55 (0.27–1.14)	0.11	11	113,975,284	5’UTR variant
**Receptor**	**HTR3A**	**rs1985242**	T > A	36	32	0.58 (0.31–1.09)	0.09	11	113,977,551	5’UTR variant
	**HTR3A**	**rs1176713**	T > C	27	22	0.74 (0.40–1.39)	0.35	11	113,989,703	Synonymous
	**HTR3B**	**rs1176744**	T > G	52	27	1.36 (0.78–2.54)	0.26	11	113,932,306	Missense
**MTHFR**	**MTHFR**	**rs1801131**^**2**^	A > C	44	34	0.77 (0.44–1.33)	0.35	1	11,794,419	Missense
**Enzyme**	**MTHFR**	**rs1801133**	C > T	49	21	1.77 (0.96–3.28)	0.07	1	11,796,321	Missense

The additive effect of the minor allele on parent-assessed MTX-induced nausea – the methotrexate intolerance severity score. Listed for every single nucleotide polymorphism (SNP) are the respective gene, the alleles, the relevant chromosome (Chr.), the genomic position, and the consequence of the SNP

*N* = 118, due to missing SNP data for two patients and MISS data for one

*OR Odds Ratio*

^1^The MTX_intolerant_ subgroup: children with a total score of the Methotrexate Intolerance Severity Score (MISS) ≥6 and at least 1 point in the anticipatory and/or associative and/or behavioral symptoms (*n* = 72). Otherwise categorised as MTX_tolerant_ (*n* = 46)

^2^SNP variant not available for one child

**Table 4:** Single nucleotide polymorphisms in association with the phenotype “MTX-nausea”

	Gene	SNP	Alleles, Major > minor	MTX-nausea^**1**^ Allel count	Controls Allel count	OR (95% CI)	p	Chr.	Genomic Position	Consequence
**Hepatic**	**ABCC2**	**rs2273697**	G > A	32	9	1.35 (0.64–2.84)	0.44	10	99,804,058	Missense
**Efflux**	**ABCC2**	**rs3740066**^**2**^	G > A	43	16	1.05 (0.48–2.30)	0.90	10	99,844,450	Missense
**Transporter**	**ABCC2**	**rs717620**	G > A	24	9	1.00 (0.43–2.34)	1.00	10	99,782,821	5’UTR variant
	**ABCB1**	**rs2032582**	G > T	45	17	0.99 (0.46–2.10)	0.94	7	87,531,302	Missense
			G > A	3	2	0.54 (0.08–3.47)	0.51			
	**ABCB1**	**rs1045642**	T > C	50	19	0.97 (0.44–2.14)	0.94	7	87,509,329	Synonymous
**Hepatic**	**SLCO1B1**	**rs4149056**	T > C	18	6	1.16 (0.41–3.31)	0.78	12	21,178,615	Missense
**Influx**	**SLCO1B1**	**rs4149081**	G > A	19	7	1.02 (0.37–2.80)	0.96	12	21,225,087	Intron variant
**Transporter**	**SLCO1B3**	**rs2117032**	T > C	43	14	1.20 (0.61–2.39)	0.60	12	20,921,188	Down stream 3’UTR
	**SLC19A1**	**rs1051266**^**2**^	G > A	48	18	1.04 (0.49–2.18)	0.93	21	45,537,880	Missense
**Nausea**	**HTR3A**	**rs1062613**	C > T	21	5	1.82 (0.60–5.47)	0.29	11	113,975,284	5’UTR variant
**Receptor**	**HTR3A**	**rs1985242**	T > A	36	9	1.85 (0.76–4.50)	0.17	11	113,977,551	5’UTR variant
	**HTR3A**	**rs1176713**	T > C	25	5	2.03 (0.73–5.60)	0.17	11	113,989,703	Synonymous
	**HTR3B**	**rs1176744**	T > G	37	14	0.99 (0.46–2.12)	0.97	11	113,932,306	Missense
**MTHFR**	**MTHFR**	**rs1801131**^**2**^	A > C	41	13	1.30 (0.62–2.71)	0.49	1	11,794,419	Missense
**Enzyme**	**MTHFR**	**rs1801133**	C > T	34	12	1.10 (0.49–2.45)	0.82	1	11,796,321	Missense

The additive effect of the minor allele on child-assessed MTX-induced nausea – the nausea diary. Listed for every single nucleotide polymorphism (SNP) are the respective gene, the alleles, the relevant chromosome (Chr.), the genomic position, and the consequence of the SNP

*N* = 77, the patients who had completed at least seven diary entries

*OR* Odds Ratio

^1^The “MTX-nausea” subgroup: children with a nausea diary illustrating a nausea pattern timely related to the MTX administration (*n* = 56). Controls (*n* = 21)

^2^SNP variant not available for one child

**Table 5:** The specific haplotypes and the phenotype “MTX-intolerance”

Chr	Markers	Haplotype	F cases	F controls	Score	***p***-value	***p***-sim
1	rs1801131-rs1801133	A-C	0.348	0.402	−0.916	0.360	0.329
1	rs1801131-rs1801133	A-T	0.340	0.228	1.852	0.064	0.06
1	rs1801131-rs1801133	C-C	0.312	0.370	−0.914	0.361	0.324
1	rs1801131-rs1801133	C-T	0.000	0.000	NA	NA	NA
7	rs1045642-rs2032582	C-A	0.021	0.022	−0.048	0.962	1
7	rs1045642-rs2032582	C-G	0.401	0.435	−0.530	0.596	0.557
7	rs1045642-rs2032582	C-T	0.008	0.000	NA	NA	NA
7	rs1045642-rs2032582	T-G	0.154	0.174	−0.432	0.666	0.694
7	rs1045642-rs2032582	T-T	0.415	0.370	0.758	0.448	0.422
10	rs717620-rs2273697-rs3740066	A-A-A	0.000	0.000	NA	NA	NA
10	rs717620-rs2273697-rs3740066	A-G-A	0.201	0.250	−0.852	0.394	0.427
10	rs717620-rs2273697-rs3740066	A-G-G	0.000	NA	NA	NA	NA
10	rs717620-rs2273697-rs3740066	G-A-A	0.023	0.018	NA	NA	NA
10	rs717620-rs2273697-rs3740066	G-A-G	0.227	0.221	0.070	0.944	0.961
10	rs717620-rs2273697-rs3740066	G-G-A	0.182	0.112	1.465	0.143	0.153
10	rs717620-rs2273697-rs3740066	G-G-G	0.367	0.399	−0.466	0.641	0.639
10	rs717620-rs2273697	A-A	0.000	0.000	NA	NA	NA
10	rs717620-rs2273697	A-G	0.201	0.250	−0.852	0.394	0.36
10	rs717620-rs2273697	G-A	0.250	0.239	0.168	0.867	0.84
10	rs717620-rs2273697	G-G	0.549	0.511	0.537	0.591	0.572
10	rs2273697-rs3740066	A-A	0.021	0.017	NA	NA	NA
10	rs2273697-rs3740066	A-G	0.229	0.223	0.110	0.913	0.936
10	rs2273697-rs3740066	G-A	0.386	0.364	0.376	0.707	0.732
10	rs2273697-rs3740066	G-G	0.364	0.397	−0.526	0.599	0.587
11	rs1176744-rs1062613-rs1985242-rs1176713	G-C-A-C	0.071	0.043	0.558	0.577	0.569
11	rs1176744-rs1062613-rs1985242-rs1176713	G-C-T-T	0.029	0.008	NA	NA	NA
11	rs1176744-rs1062613-rs1985242-rs1176713	G-T-A-T	0.220	0.156	1.153	0.249	0.265
11	rs1176744-rs1062613-rs1985242-rs1176713	T-C-A-C	0.041	0.035	0.066	0.948	0.972
11	rs1176744-rs1062613-rs1985242-rs1176713	T-C-T-T	0.000	0.051	NA	NA	NA
11	rs1176744-rs1062613-rs1985242-rs1176713	T-T-A-C	0.001	NA	NA	NA	NA
11	rs1176744-rs1062613-rs1985242-rs1176713	T-T-A-T	0.029	0.063	−1.030	0.303	0.287
11	rs1176744-rs1062613-rs1985242	G-C-A	NA	0.016	NA	NA	NA
11	rs1176744-rs1062613-rs1985242	G-C-T	0.513	0.496	0.406	0.685	0.693
11	rs1176744-rs1062613-rs1985242	G-T-A	0.048	0.098	−1.430	0.153	0.153
11	rs1176744-rs1062613-rs1985242	T-C-A	0.034	0.034	−1.283	0.199	0.142
11	rs1176744-rs1062613-rs1985242	T-C-T	0.016	NA	NA	NA	NA
11	rs1176744-rs1062613-rs1985242	T-T-A	0.097	0.054	0.822	0.411	0.397
11	rs1176744-rs1062613-rs1985242	T-T-T	0.218	0.164	1.219	0.223	0.23
11	rs1062613-rs1985242-rs1176713	C-A-C	0.045	0.075	−1.052	0.293	0.276
11	rs1062613-rs1985242-rs1176713	C-A-T	0.001	NA	NA	NA	NA
11	rs1062613-rs1985242-rs1176713	C-T-T	0.030	0.077	−1.331	0.183	0.186
11	rs1062613-rs1985242-rs1176713	T-A-C	0.516	0.488	0.355	0.722	0.735
11	rs1062613-rs1985242-rs1176713	T-A-T	0.078	0.142	−1.947	0.052	0.054
11	rs1062613-rs1985242-rs1176713	T-T-T	0.015	NA	NA	NA	NA
11	rs1176744-rs1062613	G-C	0.099	0.106	−0.190	0.849	0.862
11	rs1176744-rs1062613	G-T	0.028	0.025	0.094	0.925	0.982
11	rs1176744-rs1062613	T-C	0.734	0.652	1.486	0.137	0.129
11	rs1176744-rs1062613	T-T	0.088	0.133	−1.048	0.295	0.302
11	rs1062613-rs1985242	C-A	0.034	0.084	−1.688	0.091	0.126
11	rs1062613-rs1985242	C-T	0.016	0.000	NA	NA	NA
11	rs1062613-rs1985242	T-A	0.323	0.224	1.489	0.137	0.137
11	rs1062613-rs1985242	T-T	0.038	0.070	−0.848	0.396	0.379
11	rs1985242-rs1176713	A-C	0.538	0.559	−0.078	0.937	0.953
11	rs1985242-rs1176713	A-T	0.101	0.148	−1.663	0.096	0.098
11	rs1985242-rs1176713	T-C	0.128	0.130	−0.136	0.892	0.868
11	rs1985242-rs1176713	T-T	0.734	0.652	1.488	0.137	0.126
12	rs2117032-rs4149056-rs4149081	C-C-A	0.082	0.045	0.914	0.361	0.392
12	rs2117032-rs4149056-rs4149081	C-C-G	0.000	0.000	NA	NA	NA
12	rs2117032-rs4149056-rs4149081	C-T-G	0.279	0.325	−0.596	0.551	0.554
12	rs2117032-rs4149056-rs4149081	C-T-T	0.092	0.097	0.172	0.864	0.855
12	rs2117032-rs4149056-rs4149081	T-C-A	0.014	0.022	NA	NA	NA
12	rs2117032-rs4149056-rs4149081	T-C-C	0.533	0.512	0.162	0.872	0.895
12	rs2117032-rs4149056-rs4149081	T-T-G	0.082	0.046	0.904	0.366	0.368
12	rs2117032-rs4149056	C-C	0.279	0.324	−0.593	0.553	0.566
12	rs2117032-rs4149056	C-T	0.091	0.096	0.177	0.860	0.828
12	rs2117032-rs4149056	T-C	0.547	0.535	0.039	0.969	0.984
12	rs2117032-rs4149056	T-T	0.174	0.141	0.660	0.509	0.586
12	rs4149056-rs4149081	C-A	0.014	0.022	NA	NA	NA
12	rs4149056-rs4149081	C-C	0.813	0.837	−0.490	0.624	0.681
12	rs4149056-rs4149081	C-G	0.082	0.045	0.914	0.361	0.392
12	rs4149056-rs4149081	C-T	0.000	0.000	NA	NA	NA
12	rs4149056-rs4149081	T-C	0.279	0.325	−0.596	0.551	0.554
12	rs4149056-rs4149081	T-G	0.092	0.097	0.172	0.864	0.855
12	rs4149056-rs4149081	T-T	0.014	0.022	NA	NA	NA

The haplotype-specific scores for the listed markers in association with the parent-assessed MTX-induced nausea – the methotrexate intolerance severity score

*N* = 118, due to missing SNP data for two patients and MISS data for one

*Chr Chromosome. Markers; the SNPs in the haplotype. Haplotypes; the alleles for the SNPs in the haplotype. Cases; MTX*_*intolerant*_ *(n = 72). Controls; MTX*_*tolerant*_ *(n = 46). F*_*cases*_*;* Frequency of the haplotype among cases*, F*_*controls*_*;* Frequency of the haplotype among controls. Score; the haplotype association test score for the specific haplotype. *P*-sim the number of times the simulated score statistics exceeds the observed divided by the total number of simulations (1000)

**Table 6:** The specific haplotypes and the phenotype “MTX-nausea”

Chr	Markers	Haplotype	F cases	F controls	Score	***p***-value	***p***-sim
1	rs1801131-rs1801133	A-C	0.321	0.405	−1.067	0.286	0.333
1	rs1801131-rs1801133	A-T	0.304	0.286	0.222	0.825	0.837
1	rs1801131-rs1801133	C-C	0.376	0.310	0.730	0.465	0.523
1	rs1801131-rs1801133	C-T	0.000	0.000	NA	NA	NA
7	rs1045642-rs2032582	C-A	0.027	0.048	NA	NA	NA
7	rs1045642-rs2032582	C-G	0.420	0.405	0.184	0.854	0.929
7	rs1045642-rs2032582	C-T	0.000	0.000	NA	NA	NA
7	rs1045642-rs2032582	T-G	0.152	0.143	0.144	0.886	0.917
7	rs1045642-rs2032582	T-T	0.402	0.405	−0.035	0.972	0.931
10	rs717620-rs2273697-rs3740066	A-A-A	0.000	NA	NA	NA	NA
10	rs717620-rs2273697-rs3740066	A-A-G	0.214	0.214	0.000	1.000	0.964
10	rs717620-rs2273697-rs3740066	A-G-A	0.000	NA	NA	NA	NA
10	rs717620-rs2273697-rs3740066	G-A-A	0.014	0.000	NA	NA	NA
10	rs717620-rs2273697-rs3740066	G-A-G	0.272	0.214	0.735	0.463	0.478
10	rs717620-rs2273697-rs3740066	G-G-A	0.159	0.167	−0.011	0.991	0.977
10	rs717620-rs2273697-rs3740066	G-G-G	0.341	0.405	−0.802	0.423	0.385
10	rs717620-rs2273697	A-A	0.000	0.000	NA	NA	NA
10	rs717620-rs2273697	A-G	0.214	0.214	0.000	1.000	0.94
10	rs717620-rs2273697	G-A	0.286	0.214	0.783	0.434	0.44
10	rs717620-rs2273697	G-G	0.500	0.571	−0.748	0.454	0.469
10	rs2273697-rs3740066	A-A	0.013	0.000	NA	NA	NA
10	rs2273697-rs3740066	A-G	0.273	0.214	0.706	0.480	0.534
10	rs2273697-rs3740066	G-A	0.376	0.381	−0.014	0.989	0.966
10	rs2273697-rs3740066	G-G	0.338	0.405	−0.791	0.429	0.397
11	rs1176744-rs1062613-rs1985242-rs1176713	G-C-A-C	0.070	0.035	0.589	0.556	0.552
11	rs1176744-rs1062613-rs1985242-rs1176713	G-C-T-T	0.030	NA	NA	NA	NA
11	rs1176744-rs1062613-rs1985242-rs1176713	G-T-A-T	0.144	0.288	−1.331	0.183	0.171
11	rs1176744-rs1062613-rs1985242-rs1176713	T-C-A-C	0.060	NA	1.641	0.101	0.076
11	rs1176744-rs1062613-rs1985242-rs1176713	T-C-T-T	0.026	0.010	NA	NA	NA
11	rs1176744-rs1062613-rs1985242-rs1176713	T-T-A-C	0.035	0.064	−0.373	0.709	0.78
11	rs1176744-rs1062613-rs1985242-rs1176713	T-T-A-T	0.000	0.024	NA	NA	NA
11	rs1176744-rs1062613-rs1985242	G-C-A	NA	0.000	NA	NA	NA
11	rs1176744-rs1062613-rs1985242	G-C-T	0.534	0.470	0.151	0.880	0.87
11	rs1176744-rs1062613-rs1985242	G-T-A	0.059	0.020	0.943	0.346	0.38
11	rs1176744-rs1062613-rs1985242	T-C-A	0.042	0.060	0.086	0.931	0.917
11	rs1176744-rs1062613-rs1985242	T-C-T	NA	0.0282	NA	NA	NA
11	rs1176744-rs1062613-rs1985242	T-T-A	0.098	0.034	1.044	0.297	0.281
11	rs1176744-rs1062613-rs1985242	T-T-T	0.144	0.299	−1.300	0.194	0.197
11	rs1062613-rs1985242-rs1176713	C-A-C	0.089	0.000	1.273	0.203	0.234
11	rs1062613-rs1985242-rs1176713	C-T-C	0.036	0.089	−0.798	0.425	0.455
11	rs1062613-rs1985242-rs1176713	C-T-T	0.535	0.459	0.128	0.898	0.898
11	rs1062613-rs1985242-rs1176713	T-A-C	0.099	0.091	0.893	0.372	0.4
11	rs1062613-rs1985242-rs1176713	T-A-T	NA	0.028	NA	NA	NA
11	rs1062613-rs1985242-rs1176713	T-T-T	0.104	0.099	0.184	0.854	0.864
11	rs1176744-rs1062613	G-C	0.030	0.025	NA	NA	NA
11	rs1176744-rs1062613	G-T	NA	0.000	NA	NA	NA
11	rs1176744-rs1062613	T-C	0.679	0.757	−1.075	0.282	0.255
11	rs1176744-rs1062613	T-T	0.119	0.020	1.760	0.078	0.09
11	rs1062613-rs1985242	C-A	0.068	0.070	−0.053	0.958	0.997
11	rs1062613-rs1985242	C-T	0.000	0.028	NA	NA	NA
11	rs1062613-rs1985242	T-A	0.252	0.333	−0.579	0.563	0.564
11	rs1062613-rs1985242	T-T	0.078	0.000	1.293	0.196	0.162
11	rs1985242-rs1176713	A-C	0.560	0.548	−0.264	0.792	0.802
11	rs1985242-rs1176713	A-T	0.109	0.119	0.494	0.621	0.592
11	rs1985242-rs1176713	T-C	0.134	0.124	0.256	0.798	0.826
11	rs1985242-rs1176713	T-T	0.679	0.757	−1.072	0.284	0.241
12	rs2117032-rs4149056-rs4149081	C-C-A	0.105	0.000	1.143	0.253	0.26
12	rs2117032-rs4149056-rs4149081	C-C-G	0.279	0.333	0.009	0.993	0.978
12	rs2117032-rs4149056-rs4149081	C-T-G	0.056	0.143	−0.827	0.408	0.419
12	rs2117032-rs4149056-rs4149081	T-C-A	0.009	0.024	NA	NA	NA
12	rs2117032-rs4149056-rs4149081	T-T-G	0.551	0.500	−0.041	0.968	0.968
12	rs2117032-rs4149056	C-C	0.105	0.000	1.135	0.256	0.257
12	rs2117032-rs4149056	C-T	0.279	0.333	0.011	0.991	0.96
12	rs2117032-rs4149056	T-C	0.056	0.143	−0.824	0.410	0.5
12	rs2117032-rs4149056	T-T	0.560	0.524	−0.200	0.841	0.843
12	rs4149056-rs4149081	C-A	0.161	0.143	0.285	0.776	0.617
12	rs4149056-rs4149081	C-G	0.009	0.024	NA	NA	NA
12	rs4149056-rs4149081	T-G	0.830	0.833	−0.047	0.963	0.869

The haplotype-specific scores for the listed markers in association with the child-assessed MTX-induced nausea – the nausea diary

***N*** **= 77,** the patients who had completed at least seven diary entries

*Chr;* Chromosome. *Markers; the SNPs in the haplotype. Haplotypes; the alleles for the SNPs in the haplotype. Cases; MTX*_*nausea*_ *(n = 56). Controls (n = 21). F*_*cases*_*;* Frequency of the haplotype among cases*, F*_*controls*_*,* Frequency of the haplotype among controls. Score; the haplotype association test score for the specific haplotype*. P-sim;* the number of times the simulated score statistics exceeds the observed divided by the total number of simulations (1000)

### SNPs in the MTHFR enzyme

The selected SNPs were rs1801131 and rs1801133. There was a significant association between the genotype distribution for rs1801133 and the parent-assessed phenotype (the MISS) (*p* = 0.02); but not for the child-reported phenotype (the nausea diary) (Table 2). There was no significant additive effect of the minor alleles on either phenotype for the two SNPs (Table 3 and Table 4), or any significant haplotype associations (Table 5 and Table 6).

### SNPs in the 5-HT_3_-receptor

The selected SNPs within the candidate genes were: HTR3A (rs1062613; rs1985242; rs1176713) and HTR3B (rs1176744). No significant associations were found between the genotype distribution and the phenotype subgroups (Table 2). None of these SNPs were significantly associated with the two phenotypes (“MTX-nausea” or MTX_intolerant_) with regard to an additive effect of the minor alleles (Table 3 and Table 4). No significant haplotype associations with either phenotype were found (Table 5 and Table 6).

## Conclusion

MTX-induced nausea may be influenced by SNPs in the MTHFR enzyme (rs1801133). Our data does not support an association between MTX-induced nausea and the remaining selected SNPs in genes encoding MTX-transporter proteins or the 5-HT_3_ nausea receptor.

## Data Availability

Authors can confirm that all relevant data are included in the article and its supplementary information files. The public availability of the data that support the findings of this study are restricted by the license of the Danish Data protection agency (1–16–02-429-15).

## Abbreviations

JIA: Juvenile idiopathic arthritis
SNPs: Single nucleotide polymorphisms
MISS: Methotrexate intolerance severity score
MTX: Methotrexate
MTHFR: Methylentetrahydrofolatereductase
5-HT_3_: 5-hydroxytryptamine type 3
